# Emerging roles of mechanosensitive ion channels in ventilator induced lung injury: a systematic review

**DOI:** 10.3389/fimmu.2024.1479230

**Published:** 2024-11-27

**Authors:** Gang Liu, Bin-bin Dong, Shalika Devanarayana, Rong-Chang Chen, Qi Liu

**Affiliations:** ^1^ Department of Emergency Medicine, The First Affiliated Hospital of Zhengzhou University, Zhengzhou, Henan, China; ^2^ Translational Medicine Center, The First Affiliated Hospital of Zhengzhou University, Zhengzhou, Henan, China; ^3^ School of International Education, Zhengzhou University, Zhengzhou, Henan, China; ^4^ Guangzhou Institute of Respiratory Health, First Affiliated Hospital of Guangzhou Medical University, Guangzhou, Guangdong, China; ^5^ Department of Shenzhen Institute of Respiratory Diseases, Shenzhen People’s Hospital, Shenzhen, Guangdong, China

**Keywords:** ventilator induced lung injury, mechanosensitive ion channels, mechanical ventilation, pyroptosis, Piezo

## Abstract

**Background:**

The pathogenetic mechanisms of ventilator-induced lung injury (VILI) still need to be elucidated. The mechanical forces during mechanical ventilation are continually sensed and transmitted by mechanosensitive ion channels (MSICs) in pulmonary endothelial, epithelial, and immune cells. In recent years, MSICs have been shown to be involved in VILI.

**Methods:**

A systematic search across PubMed, the Cochrane Library, Web of Science, and ScienceDirect was performed from inception to March 2024, and the review was conducted in accordance with PRISMA guidelines. The potential eligible studies were evaluated by two authors independently. Study characteristics, quality assessment, and potential mechanisms were analyzed.

**Results:**

We included 23 eligible studies, most of which were performed with murine animals *in vivo*. At the *in vitro* level, 52% and 48% of the experiments were conducted with human or animal cells, respectively. No clinical studies were found. The most reported MSICs include Piezo channels, transient receptor potential channels, potassium channels, and stretch-activated sodium channels. Piezo1 has been the most concerned channel in the recent five years. This study found that signal pathways, such as RhoA/ROCK1, could be enhanced by cyclic stretch-activated MSICs, which contribute to VILI through dysregulated inflammation and immune responses mediated by ion transport. The review indicates the emerging role of MSICs in the pathogenesis of VILI, especially as a signal-transmitting link between mechanical stretch and pathogenesis such as inflammation, disruption of cell junctions, and edema formation.

**Conclusions:**

Mechanical stretch stimulates MSICs to increase transcellular ion exchange and subsequently generates VILI through inflammation and other pathogeneses mediated by MSICs signal-transmitting pathways. These findings make it possible to identify potential therapeutic targets for the prevention of lung injury through further exploration and more studies.

**Systematic review registration:**

https://inplasy.com/inplasy-2024-10-0115/, identifier INPLASY2024100115.

## Introduction

Currently, mechanical ventilation (MV) is one of the most important supportive options in multiple departments, such as emergency departments, operating rooms, and intensive care units, with the technical development of ventilation philosophy and ventilators (1, 2). However, MV itself can cause concomitant lesions, such as ventilator induced lung injury (VILI), which is prone to occur in patients with acute respiratory distress syndrome (ARDS) (3) due to the unique pathophysiological characteristics of the “baby lung” (4). The lung protective MV strategy has improved the prognosis of patients with ARDS to a certain extent (5–7); however, the mortality rate for individuals diagnosed with ARDS is still up to 30% to 40% (6, 8, 9). To a certain extent, VILI is an important influencing factor for prognosis (6, 10). The mechanism of VILI still needs to be elucidated, although a few doctrines, such as volutrauma, atelectrauma, among biotrauma have been proposed (11–13). The underlying commonality behind these findings might be dysregulated mechanotransduction in the lung since respiration movement itself is a mechanical process in both spontaneous breathing and supporting conditions with artificial ventilation.

The mechanical force during respiration can be sensed and transmitted by the mechanosensitive ion channels (MSICs), which are ubiquitously expressed throughout the human organs and tissues (14–16). In the lung, MSICs are found in endothelial, epithelial, and immune cells (17–20), which continually sense and withstand mechanical forces such as hydrostatic pressure, stress, and stretching. The mechanical force is transmitted by MSICs mainly through the force-from-lipids model or the force-from-tether model (14). Physiologically, mechanical forces irritate MSICs, activate downstream signal transduction pathways, stimulate lung cells to respond adaptively, and regulate lung development. Therefore, MSICs play an important role in maintaining lung homeostasis through transmembrane ions, solutes, and water transport (21). However, if the mechanical stimuli are too strong to exceed the physiological limits, inflammation process can be launched, causing an imbalance in the fluid inside and outside the cell membrane, resulting in lung injury. In recent decades, an increasing number of publications have indicated that MSICs participate in the pathophysiological alterations in the respiratory system (22–24), and even take part in variety of human lung diseases such as ARDS (22, 25), pulmonary hypertension (26), cystic fibrosis (27), lung cancer (28) and various other respiratory diseases (21, 29).

Lung diseases, especially ARDS, change lung mechanics dramatically. In patients suffering from ARDS, the heterogeneous pathological changes in the lung and the stress raiser in the margins between the atelectatic and aerated regions increase the susceptibility of the lungs to mechanical stretching during MV, and make the lung predispose to VILI (30). The initiation of VILI is attributed to the mismatching of mechanical forces acting on abnormal respiratory mechanics in vulnerable lungs (31–33). Mechanical stretch during artificial ventilation is usually unphysiological to the diseased lungs, and this unphysiological stretch aberrantly activates MSICs, induces dysregulated mechanotransduction and causes VILI. The finding that gadolinium, a pan inhibitor of MSICs, could prevent the increased lung permeability induced by high airway pressure ventilation suggested that MSICs are involved in the development of VILI (34). However, the roles of MSICs in sensing and transferring mechanical forces to biological signals, and their involvement in causing VILI, remain uncharted (11, 35). In this systematic review, we provide an overview of studies focusing on the roles of MSICs in the pathogenesis of VILI caused by positive-pressure MV.

## Methods

The study was performed complying with the PRISMA guidelines (36), and the prespecified protocol was registrated in the website of international platform of registered systematic review and meta-analysis protocols (INPLASY2024100115). Ethical review is waived because patients and animals in this study are not involved.

### Search strategy

Two researchers (G.L. and B.D.) independently performed the electronic search using databases including PubMed, the Cochrane Library, Web of Science, Embase, and ScienceDirect, for relevant articles published between the period from the inception to 2024 March. The following combinations of terms or keywords were used: (Mechanosensitive Ion Channel OR Piezo OR TRP channel OR Potassium channel OR sodium channel OR epithelial Na+ channel) AND (Ventilation Induced Lung Injury OR Ventilator Induced Lung Injury). Duplicate publications were identified and counted once. The references of the relevant publications were verified manually to identify potentially eligible studies.

### Eligibility criteria and study selection

Inclusion criteria include (1): Any study focused on the role of MSICs in ventilation induced lung injury independent of animal, cell or tissue; (2) The lung injury model was induced by mechanical ventilation or stretch *in vivo*, ex vivo or *in vitro*; (3) Original study with full-text article could be obtained. Exclusion criteria include:(1) studies published as an abstract or meeting paper without full text; (2) non original research, such as guidelines, case reports, thesis, reviews, and editorials; (3) the studies adopting multi-hit animal model, which caused the function of MSICs could not be set apart from the experiments. Firstly, the authors, G.L. and B.D., assessed all articles individually according to inclusion and exclusion criteria based on the titles and abstracts, and then judged the final qualification, inclusion or not through the full-texts. They resolved the disparities by discussion and sought a ruling from another author (QL).

### Quality assessment

The Collaborative Approach to Meta-Analysis and Review of Animal Data from Experimental Studies 10-item checklist (CAMARADES) (37) was adopted with minor modification to estimate the bias of the included studies, The items are as follows: peer-reviewed publication, control of temperature, random allocation to treatment or control, blinded induction of model, blinded assessment of outcome, use of anesthetic without significant effect on the lung injury, appropriate animal model or cells, sample size calculation, compliance with animal welfare regulations, and statement of potential conflict of interests. Each item was given one star if the specific study was qualified, otherwise, the star would be deprived; for the item “compliance with animal welfare regulations”, “unclear” was awarded if the ethical review was not mentioned owing to the requirement that all animal experiments should follow the relevant ethics in theory; Since the core mechanism of VILI is inflammation, we chose pulmonary inflammation as an indicator to assess whether anesthetics affected the effect of interventional strategy on the outcomes of interest. In experiments for VILI involving rodents, pentobarbitone was the most used anesthetic. Given that no studies reported pentobarbitone itself causing significant inflammatory reactions, we assessed that the use of anesthetics had no significant impact on lung inflammation during the short duration of anesthesia required for research on VILI. Two investigators (G.L. and B.D.) independently evaluated the study’s quality and divergences were well settled through consulting with the correspondence author.

## Result

**Figure 1:**
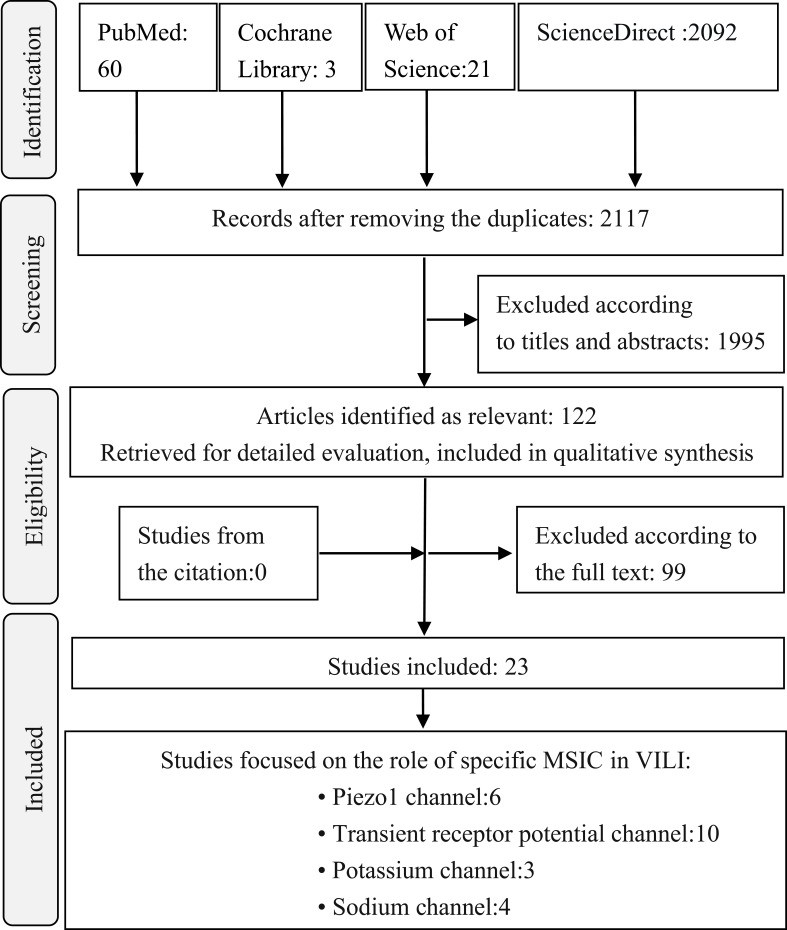
Flow chart of selection process in this systematic review. MSIC, Mechanosensitive ion channel; VILI, ventilator induced lung injury.

**Figure 2:**
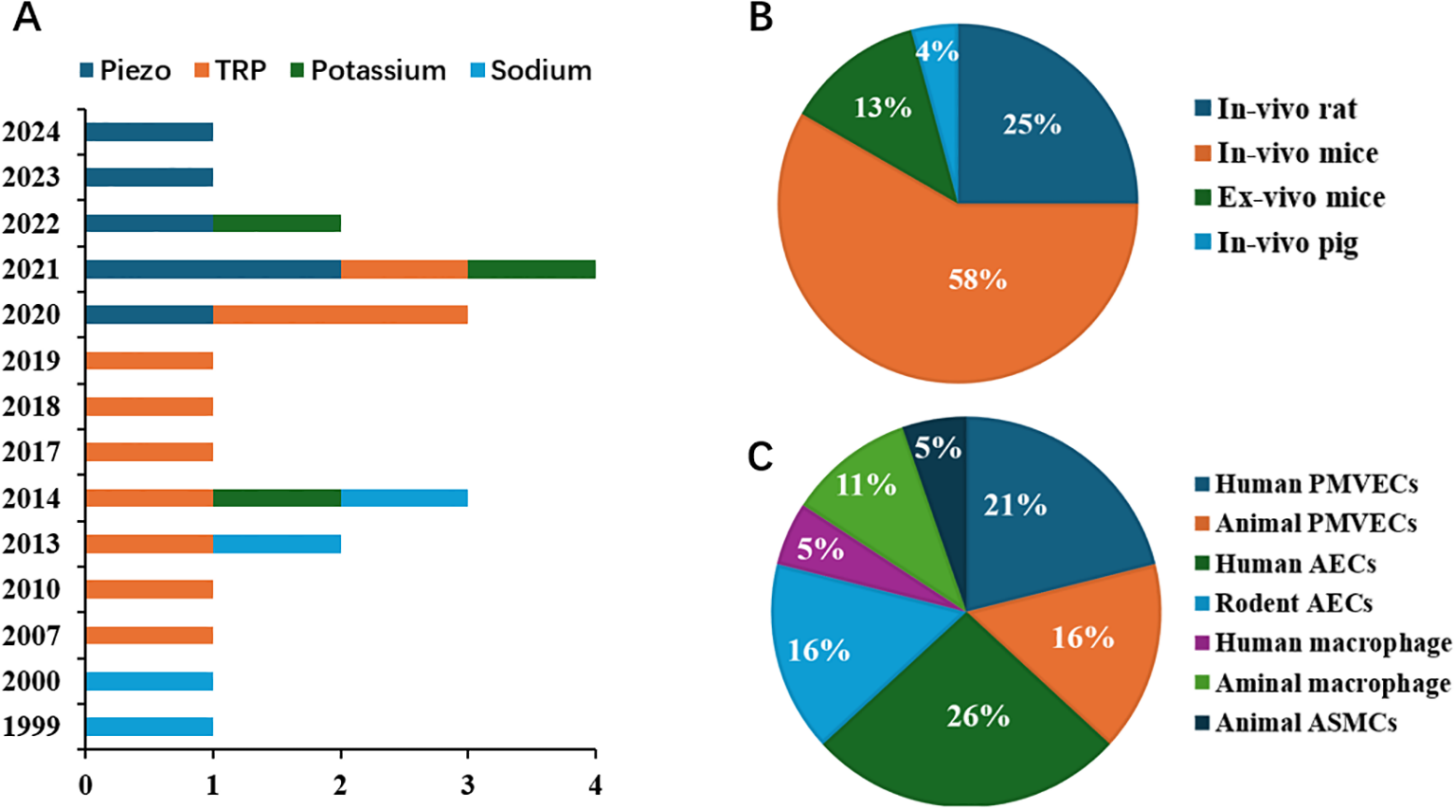
Distribution characteristics of the studies focused on the role of MSICs in VILI **(A)** Publishing timeline of studies on the role of MSICs in VILI; **(B)** Relative number of studies with various animal models on the role of MSICs in VILI; **(C)** Relative number of studies with various cells on the role of MSICs in VILI.TRP, transient receptor potential; PMVECs, pulmonary microvascular endothelial cells; AECs, alveolar epithelial cells; ASMCs, airway smooth muscle cells; MSICs, mechanosensitive ion channel; VILI, ventilator induced lung injury.

There are 2117 publications being found after removing the overlapping recordings. According to the title, abstract, and full-text, we totally found twenty-three publications qualified to be included during 1999 and 2024 March (**Figure 1**). Six studies focused on Piezo 1 channel in recent five years, transient receptor potential (TRP) channels were concerned for more than a decade five years ago (**Figure 2A**). Most of the studies were performed with murine animals (**Figure 2B**) *in vivo* level, while *in vitro* level, human AECs and PMEVECs were most adopted (**Figure 2C**). The pathogenesis was concentrated mainly on inflammation, disruption of cell junctions, edema formation, oxidative stress, cell death and signal mechanotransduction. More characteristics were summarized in **Table 1** and displayed in **Figure 2**. The number of stars ranged from 3 to 9 with a median 5 and inter quartile range 3, which indicated a medium overall quality rating (**Table 2**).

**Table 1:** Characteristics of the included studies.

Author year	Animals and models♀	Cells	Antagonist/blocking	Agonist	Signal pathway	Main pathogenesis
Piezo 1						
Zhang 2021	SD, male, rats	Human A549 AECs, human PMVECs	Piezo1: siRNA, GSMTx4; RhoA: fasudil, ROCK: Y27632	Piezo1:Yoda1	Piezo1/RhoA/ROCK1	Inflammation, apoptosis and signal transduction
Jiang 2021	Wistar, male, rats, two hit model with MS and LPS	Human PMVECs (HULEC-5a)	Piezo1: AAV-shPiezo1 (*in vivo*), siPiezo1 (*in vitro*); Calpain:Z-LLY-FMK	Yoda1	Piezo1/calpain/VE-cadherin junction complexes	Inflammation, cell junction, vascular permeability and signal transduction
Zhong 2020	Mice	Human PMVECs	Piezo1: gene deletion, GsmTx-4; Src: PP2; calpain:PD160505	Piezo1:Yoda1; Calpain: A23187	Piezo1/calpain/Src/VE-cadherin junctions	Inflammation, cell junction, vascular permeability and signal transduction
Grannemann 2023	C57BL/6 N, female, mice	Human adenocarcinoma-derived AECs, NCIH441	Piezo1: salvianolic acid B, PMA: BIM2	Piezo1:Yoda1; ADAM17: PMA, ionmycin	Piezo1/metalloproteinases (ADAM10 and ADAM17)/amphiregulin/junctional adhesion molecule	posttranslational modification of cell membrane proteins and cell junction
Fang 2022	Male, mice, two hit model with MS and hydrogen chloride	Human AECs	Piezo1: gene knockout, GsMTx4, ATP-hydrolyzing enzyme apyrase	Piezo1:Yoda1, ATP	Piezo1/ATP	Pulmonary fibrosis, epithelial mesenchymal transition and signal transduction
Luo 2024	C57BL/6, mice	Mouse airway smooth muscle cells	Piezo1: siRNA; Piezo1 knockin; ROCK: Y27632; myosin II: blebbistatin	Piezo1: eGFP	Piezo1/ROCK/integrin/migration of ASMCs	Airway remodeling, cell junction and signal transduction
TRP						
Hamanaka 2007	C57BL/6, male, mice, ex vivo	None	TRPV4: gene knockout, ruthenium red; P450 epoxygenase: miconazole; arachidonic acid: methanandamide;		TRPV4/Ca/P450/epoxyeicosatrienoic acids	acute vascular permeability
Yu 2020	C57BL/6, male, mice	Murine PMVECs	Adipose-derived exosome, TRPV4: HC-067047	TRPV4: GSK1016790A	Adipose-derived exosomes/TRPV4/Ca^2^ signaling pathway	Protective, inflammation and cell junction
Michalick 2017	C57BL/6J, male, mice, *in vivo* and ex vivo	Human PMVECs	TRPV4:gene deficient, HC-067047, rabbit anti-pSer824; SGK1: GSK650394		SGK 1/TRPV4/Ca^2^	Phosphorylation, inflammation and vascular hyperpermeability and edema formation
Hamanaka 2010	C57BL/6, male, mice	Mouse alveolar macrophage		TRPV4:4αPDD	TRPV4/Ca^2^/oxidative and nitriding stress	Oxidative stress, Nitriding stress, permeability and edema
Jurek 2014	C57BL/6, male, mice, ex vivo	Alveolar macrophages; bovine capillary endothelial cells	Nanoparticles Releasing Ruthenium Red, siRNA	TRPV4:4αPDD TRPV2: LPS; TRPVM: hydrogen peroxide	TRPV4/Ca2	lung vascular function, vascular permeability and lung edema
Pairet 2018	Balb/c, female, mice	Human AECs (NCI-H292), Human macrophages	GSK2193874, ruthenium red	GSK1016790A	TRPV4/Ca2	Inflammation
Yu 2022	C57BL/6, mice	PMVECs	TRPV4: HC-067047; ROCK: Y27632	TRPV4:GSK1016790A ROCK: calpeptin	TRPV4/ROCK1	Protective, inflammation and cell junction
Lu 2021	C57BL/6, male, mice	Ovine PMVECs	decoy peptide	TRPV4:4αPDD	TRPV4/Ca2/eNOS/mitochondrial bioenergetics	Phosphorylation, oxidative stress, inflammation and vascular permeability
TRPA1						
Wang 2013	SD, male rats	None	HC-030031		TRPA1/substance P	Inflammation and oxidative stress
TRPM2						
An 2019	C57BL/6, mice	Alveolar macrophages	TRPM2: gene knockout; NLRP3: siRNA gene knockout,SS-31; Caspase-1:siRNA, Sc siRNA, YVAD	oxidative stress: rotenone	Oxidative stress/ROS/TRPM2 and NLRP3 inflammasome	Oxidative stress, inflammation and pyroptosis
Stretch-activated potassium channel						
Roan 2014		Human A549 AECs	TREK-1: shRNA,gene deficiency;	Vinculin	TREK-1/F-actin/FAK/vinculin/cell deformability	Cytoskeletal remodeling and cell junction
Petersen 2021	Landrace/Yorkshire/Duroc, female, pigs	None	KCa3.1: senicapoc		KCa3.1/neutrophil migration	Vascular permeability and inflammation
Petersen 2022	C57BL/6, unisex, mice	Human AECs	KCa3.1: gene knockout, senicapoc		KCa3.1/neutrophil migration	Vascular permeability and inflammation
Stretch-activated sodium channels						
Lecuona 1999	SD, male rats, *in vivo* and ex vivo	Rat ATII cells			Na,K-ATPase/active sodium transport/lung edema clearance	Vascular permeability, lung edema, active sodium transport and Na,K-ATPase function in AECs
Saldias 2000	SD, male,rats	Rat ATII cells	Microtubular:colchicine; Na^+^ channel: amiloride; Na,K-ATPase: ouabain	βAR: terbutaline, isoproterenol	β-adrenergic stimulation/ion-transporting proteins/lung edema clearance	Lung edema clearance ability
Li 2013	Wistar, male, rats	None	Adrenergic receptor: prazosin, yohimbine, atenolol, ICI-118551; Na+ channel: amiloride; Na,K-ATPase: ouabain; microtubular: colchicine,β-lumicolchicine	βAR: phenylephrine	β-adrenergic stimulation/Na+/K+-ATPase/lung edema clearance	Na+/K+-ATPase activity and lung edema clearance ability
Li 2014	Mice	Mouse AECs	Claudin 18: ENaCs, amiloride; βAR: propranolol CFTR: CFTR(inh)- 172	βAR: terbutaline	Claudin 18/βAR/CFTR, ENaC, and Na-K-ATPase	Cell junction, alveolar barrier and fluid homeostasis

♀Unless otherwise specified, animals in **Table 1** are one hit models through mechanical ventilation; SD, Sprague–Dawley; MS, mechanical stretch; AEC, alveolar epithelial cell; PMVECs, pulmonary microvascular endothelial cells; LPS, lipopolysaccharide; SGK 1,glucocorticoid–regulated kinase 1, eNOS, Endothelial nitric oxide synthase; ASMCs, airway smooth muscle cells; TRPV4, transient receptor potential vanilloid 4; TRPA1, transient receptor potential channel A1; ROS, reactive oxygen species; 4αPDD, 4-α-phorbol didecanoate; ENaCs, epithelial sodium channels; βAR, b-adrenergic receptor; CFTR,cystic fibrosis transmembrane conductance regulator.

**Table 2:** Quality assessment of the included studies.

Author year	Peer-reviewed publication	Control of temperature	Random allocation to treatment or control	Blinded induction of model	Blinded assessment of outcome	Use of anesthetic without significant effect on the lung inflammation	Appropriate animal model or cells	Sample size calculation	Compliance with animal welfare regulations	Statement of potential conflict of interests	Total stars
Piezo 1											
Zhang 2021	★	★	★	–	★	★	★	–	★	★	8
Jiang 2021	★	★	★	–	–	★	★	–	★	★	7
Zhong 2020	★	–	–	–	–	★	★	–	★	–	4
Grannemann 2023	★	–	–	–	–	★	★	–	★	★	5
Fang 2022	★	★	–	–	–	★	★	–	★	★	6
Luo 2024	★	★	★	–	–	★	★	–	★	★	7
TRP											
Hamanaka 2007	★	★	★	–	–	★	★	–	★	–	6
Yu 2022	★	–	★	–	★	★	★	–	★	★	7
Michalick 2017	★	–	★	–	★	★	★	★	★	★	8
Hamanaka 2010	★	–	**-**	–	–	★	★	–	★	★	5
Jurek 2014	★	★	–	–	–	★	★	–	★	★	6
Pairet 2018	★	–	–	–	–	★	★	–	★	–	4
Yu 2020	★	–	–	–	★	★	★	–	★	★	6
Lu 2021	★	★	★	–	–	★	★	–	Unclear	★	5
TRPA1											
Wang 2013	★	★	★	–	–	★	★	–	★	–	5
TRPM2											
An 2019	★	–	★	–	–	★	★	–	★	–	5
Stretch-activated potassium channel											
Roan 2014	★	★	–	–	–	–	★	–	–	–	3
Petersen 2021	★	–	★	★	★	★	★	★	★	★	9
Petersen 2022	★	★	★	★	★	★	★	★	Unclear	★	9
Stretch-activated sodium channels											
Lecuona 1999	★	–	–	–	–	★	★	–	Unclear	–	3
Saldias 2000	★	★	–	–	–	★	★	–	Unclear	–	4
Li 2013	★	–	★	–	–	★	★	–	Unclear	–	4
Li 2014	★	–	–	–	–	★	★	–	Unclear	★	4

★ the quality met the criterion of this specific item, - the item was not qualified to be awarded a star, Unclear, “unclear” was awarded if the ethical review was not mentioned owing to the requirement that all animal experiments should follow the relevant ethics in theory.

### Piezo channels and VILI

Piezo proteins were first reported in 2010, and their congeners are found in protozoa, plants, and animals (38). Piezo is the largest transmembrane nonselective cation ion channel identified so far, and it contains 114 transmembrane domains with pore-forming subunits (39–41). In vertebrates, there are two Piezo family members, Piezo 1 (Fam38A) and Piezo 2 (Fam38B) (38). In humans, Piezo channels are distributed in the cardiovascular system, respiratory system, neurons and other organs (42). Mechanical signals are sensed by Piezo proteins and transduced into biological signals by mediating Ca^2+^ influx through the gated poles of Piezo channels. In mammalian lungs, Piezo1 is mainly expressed in non-excitable cells such as endothelial cells, epithelial cells, myeloid cells, and smooth muscle cells of small arteries in the lungs, and it plays a critical role in pulmonary edema, lung cancer, lung inflammation, and pulmonary hypertension; Piezo2 is expressed in the pulmonary neuroepithelial cell body, pulmonary neuroendocrine cells, and sensory neurons being associated with respiration, such as the nodose ganglion, and dorsal root ganglion in the spinal cord. Therefore, Piezo2 might be involved mainly in overinflation of lung owing to the impaired Hering-Breuer reflex, premature neonatal death, ARDS, allergic asthma, and obstructive sleep apnea syndrome (24).

**Figure 3:**
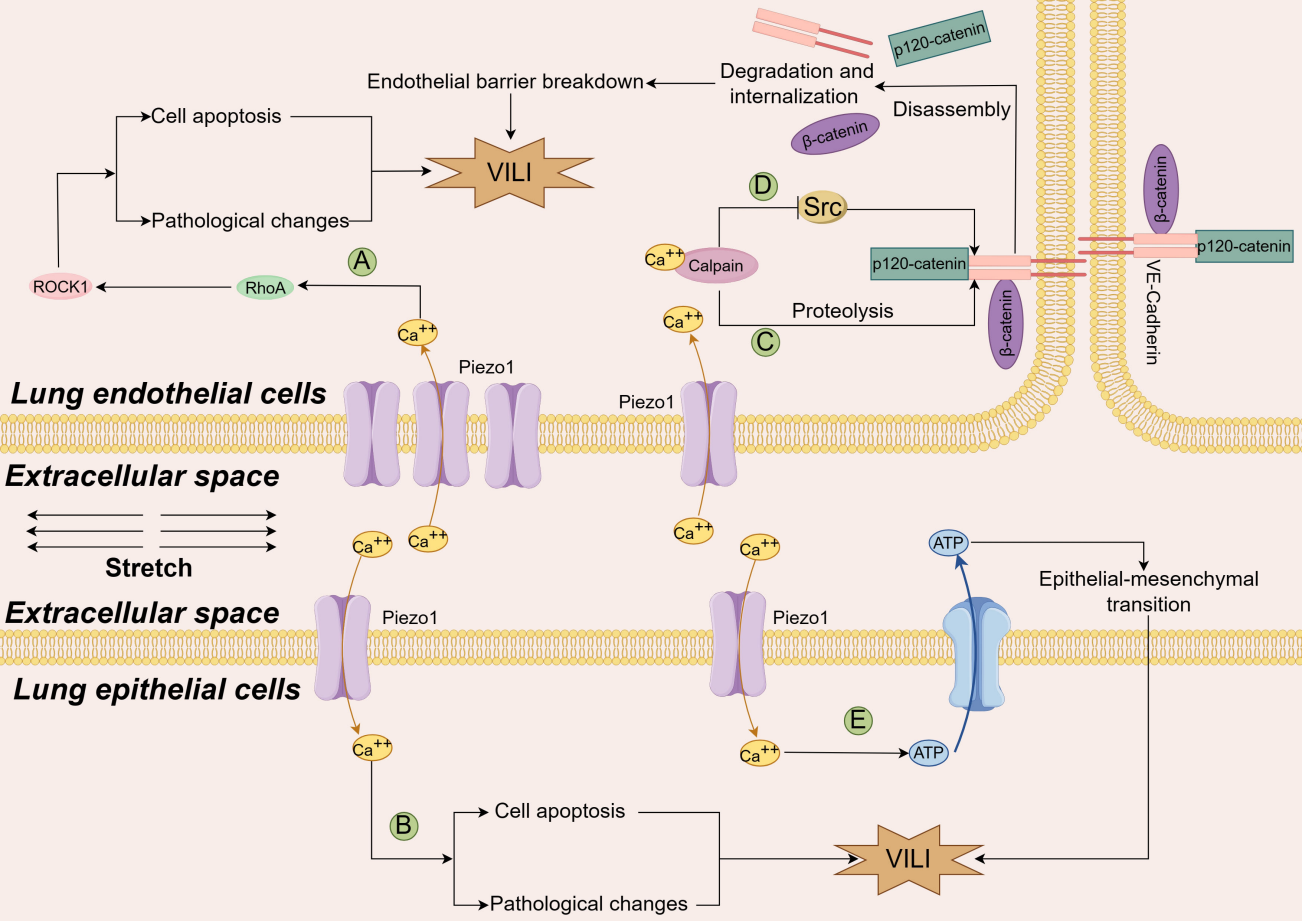
Diagram of the mechanism by which the Piezo1 channel contributes to VILI. An increase in the number of Piezo1 channels due to cyclic stretch permits more calcium ions to enter both endothelial cells **(A)** and alveolar epithelial cells **(B)**, stimulates the Rhoa/Rock1 pathway, increases cell apoptosis and exacerbates the lung pathological changes through yet undefined mechanism (Ref (44); An increase in the number of calcium ions through the stretched Piezo1 channel on one hand activates Calpain to cleave the VE-cadherin junction complexes, and disrupt the integrity of the endothelial barrier **(C)**, Ref (47). On the other hand, calcium ions activate calpain and phosphorylate Src kinase, which blocks the disassembly of endothelial adherens junctions caused by excessive mechanical stretching, Piezo1 channels play a protective role in VILI **(D)**, Ref (44) and Ref (48). Additionally, in two hit models, Piezo1 channels mediate calcium ions entry into the cells, release ATP in lung epithelial cells, promote epithelial-mesenchymal transition and lung tissue fibrosis through yet uncovered mechanisms **(E)**, Ref (50). VILI ventilation-induced lung injury, Rhoa, Ras homolog gene family member A; ROCK1, Rho-associated coiled-coil containing protein kinase 1; ATP, adenosine triphosphate; VE-cadherin, vascular endothelial cadherin.

As shown in **Figure 3**, there was no consensus on the role of Piezo proteins in VILI. Physiologically, Diem and his colleagues reported that mechanical stretch can activate the Piezo 1 channel in the caveolae of ATI cells, increase Ca^2+^ entry into cells and ATP release from ATI, ultimately stimulating surfactant secretion from ATII cells. These changes help cells adapt to mechanical stretch and protect them from injury (43). Under pathological conditions, the expression of Piezo1 can be increased by excessive stretching in both lung endothelial and alveolar epithelial cells (AECs) (44). Inhibition of Piezo1 potentially alleviated VILI, as evaluated by pathological scores, pathological changes, wet/dry weight ratios, protein leakage in the lungs, and systemic inflammation. Mechanistically, the expression of RhoA (a small GTPase protein)/Rho associated coiled-coil containing protein kinase 1 (ROCK1) could be enhanced by cyclic stretch-activated Piezo1 or a Piezo1 agonist (Yoda1); in contrast, it could be antagonized by ablation or inhibition of Piezo1. These findings indicate that Piezo1 mediates VILI through activation of the RhoA/ROCK1 signaling pathway in lung endothelial cells (44), which regulates intercellular junctions (45, 46). Similar results were reported in another study with a two-hit lung injury model. Knockout of Piezo1 strongly relieves the severity of lipopolysaccharide- induced VILI in rats with ARDS, and further experiments have shown that excessive mechanical stretching opens the Piezo1 channel, permits calcium ions to enter cells, stimulates calpain, disassembles beta-catenin, p120-catenin, and the VE-cadherin complex, induces the internalization and degradation of the VE-cadherin, breaks down the endothelial barrier, and finally induces lung injury (47).

However, Zhong et al. reported different findings. The results indicated that lung vascular permeability was greater in mice lacking the Piezo1 gene than in wild-type control mice when both were ventilated with a high tidal volume (48). Increased permeability was simultaneously detected in human monolayer lung endothelial cells that were expropriated of Piezo1, and then subjected to cyclic stretch for up to 2 hours. Further results indicated that Piezo1 deficiency in these cells destabilized VE-cadherin junctions by enhancing Src activity; and increasing VE-cadherin Tyr658 and Tyr685 phosphorylation, which caused VE-cadherin internalization. On the other hand, they reported that activation of Piezo1 and downstream Ca^2+^-dependent cysteine protease calpain could stabilize VE-cadherin junctions via reducing Src-mediated phosphorylation. They concluded that Piezo1 protected adherens junctions and maintained the integrity of the lung vascular barrier when mice received high volume MV and human lung endothelial cells were exposed to large cyclic stretch. This protective effect was attributed to Src-dependent disassembly of endothelial junctions being blocked by calpain activated by increased Ca^2+^ through the Piezo channel, after which phosphorylated calpain cleaves Src kinase (48).

The limited proteolysis is a critical posttranslational modification to guarantee the proper functions of cell membrane proteins. In the lung AECs, the a disintegrin and metalloproteinase (ADAM) family cleaves the substrate proteins expressed on AECs to make the soluble ectodomain shed from the cell membrane, and regulates the function of receptors, cytokines, growth factors, and adhesion molecules. ADAM17 and ADAM10 are the two representative ADAM proteinases. It was shown that the release of growth factor amphiregulin and junctional adhesion molecule A was enhanced through the metalloproteinases (ADAM10 and ADAM17), which was activated by stretch-triggered-Piezo1 channel in murine lungs (49). Research with a two-hit model indicated that mechanical stretching exacerbated the hydrochloric acid-induced lung injury and enhanced pulmonary fibrosis, which was driven by Piezo1-mediated ATP release in AECs (50). Another mechanism of VILI is airway remodeling caused by the migration of airway smooth muscle cells, one study found the migration of airway smooth muscle cells (ASMCs) could be enhanced by overstretch through the down-regulation of Piezo1 mRNA expression accompanied with the reduced expression of integrin αVβ1 and fewer cell adhesion of ASMCs. The enhanced migration of ASMCs could be intercepted by inhibition of ROCK signaling in the mice and ASMCs, which might assist in finding a potential intervention target to treat VILI (51).

There is no robust evidence indicating the role of Piezo2 in VILI, although Piezo2 is considered an MSIC, and the impairment of Piezo2 is associated with sleep apnea (52) and pulmonary hypertension (53).

### TRP channels and VILI

**Figure 4:**
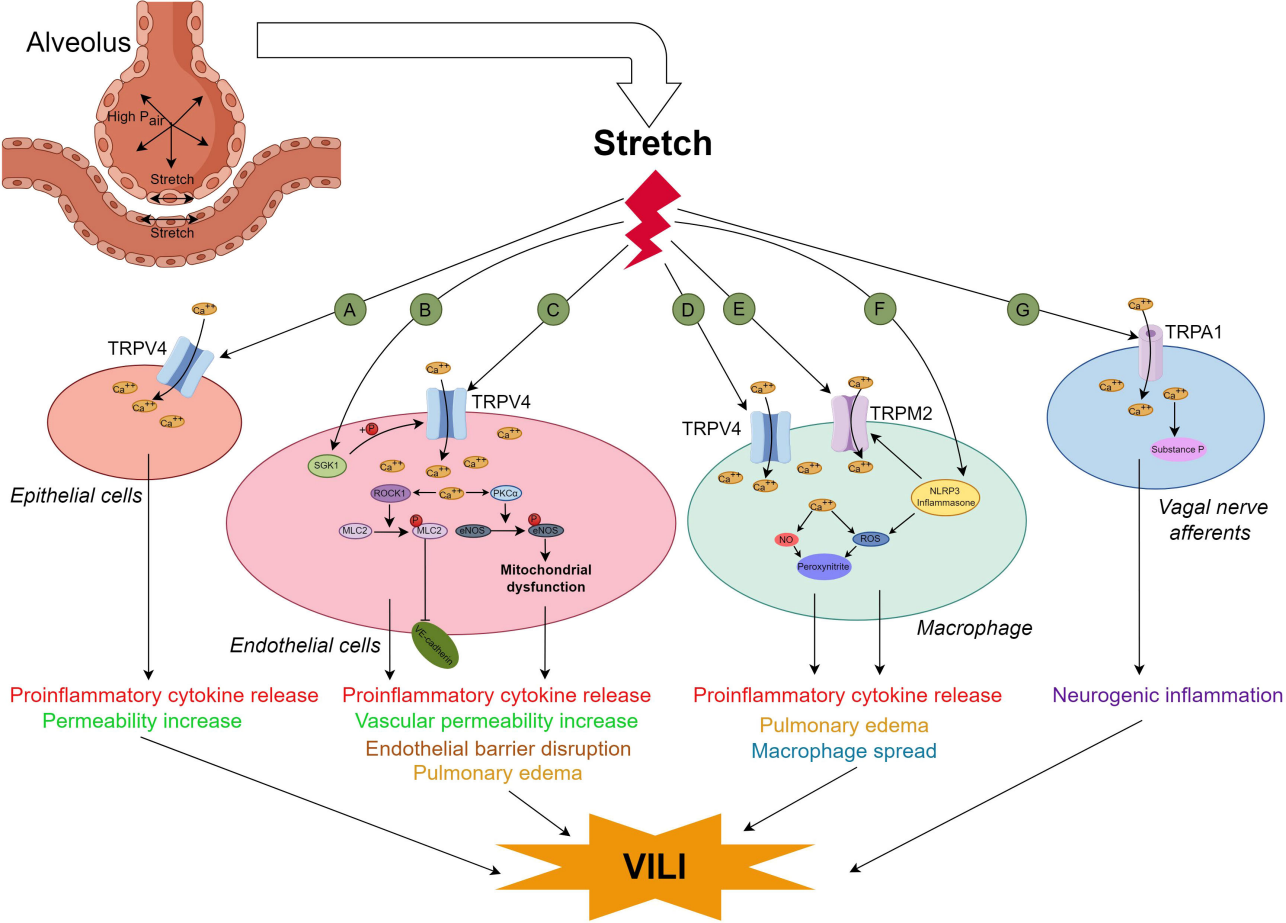
Diagram of the mechanism by which TRP channels contribute to VILI. The TRP channels related to VILI were found in alveolar epithelial cells (AECs) **(A)** Ref (64), lung endothelial cells **(B)** Ref (60); **(C)** Ref (58),Ref (59),Ref (65) and Ref (67), macrophages **(D)** Ref (62–64); **(E)** Ref (85); **(F)** Ref (85) and vagal nerve afferents **(G)**, Ref (79). When lung cells withstand mechanical stretch overload, more calcium ions enter AECs, endothelial cells and macrophages through TRVP4, provoke cytokine release and cause VILI directly or through the ROCK1/MLC2 and PKCa/eNOS pathways. In endothelial cells, the TRPV4 can also be activated by SGK1; In the lung macrophages, TRPM2 could be activated by direct stretching or though the stretch-induced NLPR3 inflammasone indirectly, the increase in intracellular calcium ions boosts more peroxynltrite through NO or ROS, promotes cytokine release, pulmonary edema and macrophage spread, and finally causes VILI; VILI can also be induced by neurogenic inflammation through TRPA1 mediated vagal nerve afferents. VILI ventilation-induced lung injury, TRP transient receptor potential, TRPV4, TRP vanilloid 4; SGK1, serum and glucocorticoid-inducible kinase 1;PKCa, protein kinase C; eNOS, endothelial nitric oxide synthase;TRMP2,transient receptor potential melastatin 2; NLRP3, NOD-like receptor family pyrin domain containing 3; ROS, reactive oxygen species;TRPA1, transient receptor potential ankyrin 1.

TRP channels were first identified in 1969 (54) and are currently known for their original name, although subsequent studies confirmed that wild-​type channel actually generates a persistent current in response to the stimulation (29). TRP channels can be activated by both mechanical and chemical stimuli, and most of the activated TRP channels regulate Ca^2+^ release in intracellular organelles (29). In the TRP superfamily, there are seven subfamilies including the TRP canonical subfamily (TRPC 1−7), vanilloid subfamily (TRPV1−6), ankyrin subfamily (TRPA1), melastatin subfamily (TRPM1−8), mucin subfamily (TRPML1−3), polycystin subfamily (TRPP/PKD), and TRPN/NompC (15, 29). As TRP channels are vital players in the mechanotransduction process, the role of TRP channel families in VILI has been explored in recent years, and the reported mechanisms are summarized in **Figure 4**.

In the TRPV subfamily, TRPV4 is the most commonly reported channel related to VILI, suggesting that it might be a potential therapeutic target for the prevention of VILI (55). TRPV4, a non-selective and polymodally gated cation channel, was first discovered in the rat kidney (56) although it is now considered to be widely expressed in various cell types across tissues including the kidneys, skin, brain, vascular tissue, lungs, and bones, and contributes to diverse functions (57). Human TRPV4, a tetrameric protein, is comprised of 871 amino acids. Each subunit of TRPV4 contains six transmembrane regions and intracellular C- and N-terminal tails (56). TRPV4 can be activated by various stimuli, including environmental factors such as mechanical stress, hypoosmotic conditions, and temperature, as well as endogenous factors like anandamide and arachidonic acid, thereby participating in a variety of physiological and pathophysiological processes through regulating the homeostasis of intracellular ion concentrations (57). Hamanaka K et al. reported that high peak inflation pressure ventilation could increase lung vascular permeability, and rapid calcium entry into endothelial cells via TRPV4 channels is the major determinant (58). It was also reported that the TRPV4/Ca^2+^ pathway was involved in the “obesity paradox” (obesity was associated with a better prognosis in patients ventilated for ARDS).The data indicated that VILI could be attenuated in mice fed a high-fat diet and in pulmonary microvascular endothelial cells treated with adipose-derived exosomes through the TRPV4/Ca^2+^ pathway (59). The effect of the TRPV4 channel/Ca^2+^ pathway in VILI might be upregulated by serum glucocorticoid-regulated kinase 1, which enhanced the phosphorylation of TRPV4 at serine 824 (60).

TRPV4 channels are also expressed in macrophages, and macrophages in alveoli are reportedly involved in the early phase of VILI (61). As a result, researchers have attempted to link TRPV4 in macrophages with VILI, and found that the activation of TRPV4 in lung macrophages could amplify the reactive nitrogen and reactive oxygen species pathways to increase the synthesis of peroxynitrite, and expedite the permeability of the lung vasculature generated by ventilation with high pressure and volume (62). VILI can be blocked by inhalation of nanoparticles with ruthenium red, a pan calcium channel blocker (63). However, the increase in proinflammatory cytokines induced by mechanical stretch was partially blocked by the selective TRPV4 inhibitor, GSK2193874, but nearly entirely blocked by ruthenium red (a blocker of pan calcium channel), which suggested that more than one calcium dependent channel is involved in the occurrence of VILI (64).

The role of ROCK1, as a cytoskeleton regulator, its role in VILI and TRVP4 is focused. One study found VILI was mediated via TRPV4/ROCK1/myosin light chain 2 signal pathway and the severity of VILI could be attenuated by lipoaspirate nanoparticles with evidence of *in vivo* and *in vitro* levels (65). Endothelial nitric oxide synthase (eNOS) uncoupling has also attracted attention because of its latent pivotal role in ARDS (66). Cyclic stretch induces Ca^2+^ influx from the TRPV4 channel into the cell, phosphorylates PKC, uncouples and redistributes eNOS at T495, increases the levels of ROS in mitochondria, decreases the mitochondrial membrane potential, disrupts mitochondrial bioenergetics, and finally causes a surplus of peroxynitrite production and increased endothelial barrier permeability (67). These changes could be mimicked by the agonist of TRPV4, 4αPDD, and phorbol myristate acetate, a PKC activator. Additionally, VILI can be alleviated by blocking eNOS phosphorylation via a decoy peptide (d-peptide) designed to restrain the phosphorylation of eNOS T495 (67). The role of the other subfamilies of TPRV in VILI still needs to be elucidated and might be promising because they are reportedly involved in ovalbumin-induced asthma models (TRPV1, TRPV2, TRPV5) (68, 69), and lung epithelial injury caused by cigarette smoke (70), LPS (71), seawater inhalation (72) and wood smoke (TRPV3) (73–75).

Some evidence suggests that VILI might be regulated by neuroimmune reactions via the cholinergic anti-inflammatory pathway (76). Ca^2+^-permeable nonselective TRPA1 cation channels are expressed in the nonneuronal cells and sensory neurons distributed on the airway and lung. Activation of TRPA1 contributes to protective neuroimmune interactions to maintain homeostasis, thermotaxis, and modulation of lifespan, and TRPA1 is involved in some respiratory diseases when TRPA1 is activated by intense harmful signals such as LPS, stress and UV light (77, 78). One study indicated that activation of TRPA1 in the vagus pathway exacerbates the neurogenic inflammation and results in VILI (79). TRPM2 (a redox-sensitive and nonselective cation channel) is distributed throughout the body and is sensitive to oxidative stress (80–82), and it modulates phagocyte ROS production (83) and neutrophil trans endothelial migration (60, 84). High tidal volume activated TRPM2 channels and the NLRP3 inflammasome, augmented oxidative stress, and ultimately promoted VILI (85). No studies have been found regarding the role of other members of the TRP channel in VILI.

### Potassium channels and VILI

Potassium channels are found in almost all living organisms and are assembled with a pore-forming domain and a regulatory domain (86). Based on their structure and function, potassium channels can be classified as inwardly rectifying, voltage-gated, tandem pore domain (K2P), ligand-gated potassium channel or potassium channel with a unitary pore-forming domain (86, 87). K^+^ ions are exclusively permitted to pass through the selective filters in the pore-forming domain. In specific cells, the function of potassium channels is regulated by the auxiliary subunits (86).

**Figure 5:**
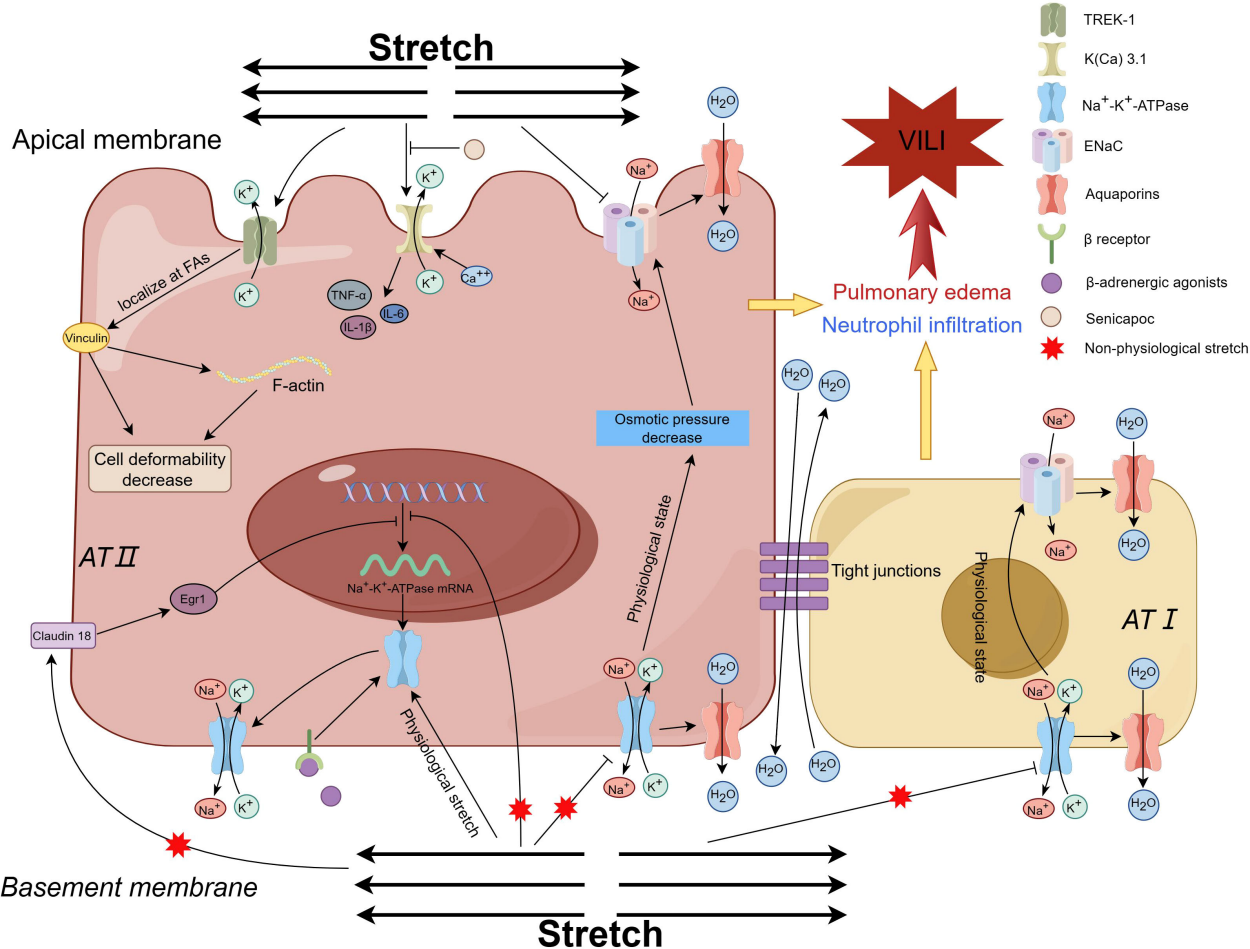
Diagram of the mechanism by which potassium channels and sodium channels contribute to VILI. Mechanical stretching activates TREK-1, leads to restructuring of the cytoskeletal framework, modulation of the deformability of AECs, and exerts an impact on cell detachment Ref (91). The inhibition of K(Ca) 3.1 channel results in the reduction of inflammation, enhancement of oxygenation and lung compliance, and mitigation of VILI, which indicates K(Ca) 3.1 channel contributes to VILI Ref (93, 94). In physiological state, ENaCs in the apical membrane regulate the alveolar fluid clearance to keep the fluid homeostasis through passive intracellular transportation of sodium under the osmotic force drive, which is produced by the active pumping Na+ ions out of the AECs through the Na-K-ATPase in the basolateral membrane. Within the physiological limits, the Na^+^-K^+^-ATPase activity is augmented to adapt to the mechanical stretch by facilitating the recruitment of Na^+^-K^+^-ATPase subunits from the intracellular pool to the basolateral membrane Ref (111, 112). Mechanical ventilation with non-physiological tidal volume weakens the Na^+^-K^+^-ATPase activity and simultaneous decreases the alveolar fluid clearance capacity Ref (109), Ref (113–115). Knockout of the Claudin 18 gene increases the expression of the Na^+^-K^+^-ATPase, and solute permeability, while decreases the sensitivity to VILI when exposed to injurious ventilation, which indicates that stretch inhibits the expression of the Na^+^-K^+^-ATPase through Claudin 18/Egr1 Ref (116). The impaired Na^+^-K^+^-ATPase activity could be restored by beta-adrenergic stimulation. VILI ventilation-induced lung injury, TREK-1 TWIK-Related K^+^ Channel 1, AECs alveolar epithelial cells, AFC alveolar fluid clearance, ENaC epithelial sodium channels. K(Ca) 3.1, calcium-activated potassium channel 3.1.

Recently, potassium channels, mainly K2P, have been reported to be associated with VILI owing to the findings that K2P might act as a mechanosensor and respond to mechanical stretch (88). There are six subfamilies (TREK, TWIK, TALK, TASK, THIK, and TRESK) of K2P channels, and they participate in diverse physiological processes (88). Three subfamilies of K2P, TREK-1, TREK-2 and TRAAK, are considered mechanosensitive channels, which can be activated by mechanical stretch and other mechanical stimuli (89). At the *in vitro* level, the K^+^ channel TREK-1 was first found to be involved in the regulation of AEC inflammation and repair (90). Further study with A549 cells indicated that mechanical stretching provoked TREK-1, remodeled the cytoskeletal structure, affected cellular detachment, and regulated the deformability of AECs through increasing fibrous actin and vinculin in focal adhesions, the effect might be relevant to the phosphorylation of focal adhesion kinase at the Tyr^925^ residue, but not at the Tyr^397^ site (91); however, the regulatory effect of TREK-1 on cytoskeletal filaments did not affect the levels of cytokines such as MCP-1or IL-6 (92). The other two subfamilies of K2P channels, TREK-2 and TRAAK, might also be involved in the lung injury caused by hyperoxia plus injurious MV (17). Petersen G and his colleagues investigated the effect of senicapoc in a porcine two-hit (lung lavage plus injurious MV) model of ARDS, and reported that the blocker of calcium-activated potassium channels (KCa3.1), senicapoc, decreased the number of neutrophils in the alveolar space and lung hemorrhage, although it did not improve oxygenation (93). A study with an one-hit model of VILI revealed that the reduction in the PaO_2_/FiO_2_ ratio, lung compliance, and TNF-α and protein levels could be prevented by pretreatment with senicapoc (94). These studies denoted that stretch-activated potassium might correlate with the development of VILI, as shown for the apical membrane in **Figure 5**.

### Stretch-activated sodium channels and VILI

Lung edema is one of the pathophysiological features of ARDS and VILI. Nowadays, sodium channel-related inflammation and lung edema are concerns in respiratory system diseases (95). Epithelial Na^+^ channels (ENaCs) are distributed in both the type I and type II AECs, and are associated with a few respiratory disease such as cystic fibrosis, obstructive lung diseases, and acute lung injury (95). The typical ENaCs are assembled anticlockwise as a heterotrimer with α, β and γ subunits in a 1:1:1 stoichiometry, which forms a mechanogated ion channel (96). In the physiological state, ENaCs in the apical membrane regulate alveolar fluid clearance (AFC) to keep the fluid homeostasis through passive intracellular transport of sodium under osmotic force drive (23, 97, 98), which is produced by the active pumping of Na^+^ ions out of AECs through the Na-K-ATPase in the basolateral membrane (99–102). The function of ENaCs is partly affected by the chloride channels, which are expressed in the lungs and are associated with the occurrence of airway viral infections, pulmonary arterial hypertension, and cystic fibrosis (103). In addition to being directly activated by mechanical stretch itself, ENaCs can also be activated via a tethering mechanism or stretch-mediated inflammatory molecules (23). The α-subunit of ENaC is a critical factor of AFC (104), and its expression during the fetal development period could be enhanced by physiological mechanical stretch via the p38 MAPK and c-Jun NH2-terminal kinase pathways (105). Overload mechanical stretch provokes the release of mediators, which impair the transepithelial Na^+^ transport function of ENaCs and induce the development of lung edema (102, 106, 107).

The transepithelial transport of solutes and water through ENaCs on the apical side highly depends on the Na^+^- K^+^-ATPase activity on the basolateral side. In humans, Na^+^- K^+^-ATPase contains four α-subunit isoforms and three β-subunit isoforms, and is usually also related to γ-subunits, which transport Na^+^ and K^+^ in the cell to maintain transmembrane gradients (100, 108). Damage to Na^+^- K^+^-ATPase activity indirectly weakens the function of ENaCs and hence induces lung injury. Lecuona, et al. ventilated a rat lung model with a high tidal volume (peak airway pressure of approximately 35 cm H_2_O) and reported that a high tidal volume impaired the ability of AFC compared with that of the control groups (moderate Vt, low Vt and nonventilated), and the impairment of AFC parallelled with a decrease in basolateral Na^+^- K^+^-ATPase activity, that is, a decrease in active sodium transport (109) (basolateral membrane in **Figure 5**). The impaired AFC could be restored by beta-adrenergic stimulation (110, 111), which could enhance the activity of both the apical ENaC and the basolateral Na^+^- K^+^-ATPase in AECs by promoting the recruitment of ion-transporting proteins, similar to those of AECs being stretched by physiological stimuli (110). At the physiological level, mechanical stretch might be harmless, and studies with murine AECs have shown that short-term (30 to 60 min) cyclic stretch during physiological elongation increases Na^+^- K^+^-ATPase activity by both augmenting intracellular Na^+^ and enhancing the recruitment of Na^+^-K^+^-ATPase subunits from intracellular pools to the basolateral membrane (112, 113). The VILI induced by nonphysiological mechanical ventilation can be alleviated by the overexpression of the β1 subunit (114) or α2 subunit of Na^+^- K^+^-ATPase (115), which increases AFC. On the other hand, the AFC ability was sharply decreased by knocking out the β1 subunit, and the residual AFC ability might be maintained mainly by a compensatory increase in the β3 subunit (116) (AT I AECs in **Figure 5**). This evidence indicates that the β1 and α2 subunits of the Na^+^- K^+^-ATPase complex mainly affect the AFC and VILI.

The activity of Na^+^-K^+^-ATPase could also be affected by tight junctions. Claudin 18 is abundant in the AECs and determines the tightness of tight junctions. Knocking out the gene of claudin 18 would increase the solute permeability, but the water content (measured as wet-to-dry ratio) in lung tissue did not increase, moreover, compared with the wild-type mice, claudin 18 knockout mice presented decreased sensitivity to VILI when they received injurious MV. Further experiments indicated a 2.3 fold increase in Na^+^- K^+^-ATPase activity in the knocking out mice and the level of β1 subunit increased up to 61% compared with that in wild-type mice (117) (**Figure 5**). Compared with that of cyclic stretch, the effect of static stretch on AECs is different, although it could also cause Na^+^-K^+^-ATPase trafficking in the basolateral membrane (118). Static stretch mainly induces cell death and remodeling of cytoskeletal organization, which is affected both by the extent of the stretch and by the stretch time. In pathophysiology, AEC remodeling makes the lung tissue insensitive to mechanical stimuli and helps the lung “escape” from unphysiological stretch (118). Additionally, the voltage-sensitive Na channel (Nav1.5–1.9), which expressed in the airways including bronchial smooth muscle cells and afferent nerve endings, can also be activated by mechanical stretching (23). However, the role of voltage-sensitive Na channels in VILI has not been reported, although the activation of these channels by mechanical stretching is associated with brain trauma (119).

## Discussion

This study is the first systematic review to exclusively explore the roles of MSICs in VILI. Substantial advances have greatly deepened the recognition in the structure and function of MSICs as well as its emerging roles in the pathogenesis of VILI over the past decades. Additionally, the findings have paved the way for excavating the potential clinical therapeutic targets. In fact, some preclinical and clinical trials have been preliminarily conducted.

Due to the relatively short history of researching Piezo channels, there are currently no clinical studies focusing on their ligands’ clinical effects although they are regarded as potential therapeutic targets (120) and blockers have been developed (121). Clinical studies involving patients still lies at the level of describing the levels of Piezo gene expression and protein in certain diseases mainly aiming to elucidate the pathogenesis mechanism (50, 122). Numerous TRPV4 antagonists have been developed and patented in recent years (123). Clinical trials have mainly focused on GSK2798745,which has been shown to be safe and effective in improving edema associated with heart failure and coronavirus disease 2019 (COVID-19) in several clinical studies although small patient pools were recruited (123). HC-030031 is a TRPA1 antagonist that holds promise for entering clinical research (124). Although K2P is considered a potential therapeutic target, it has not yet reached the stage of clinical or preclinical trial (125). Senicapoc, a KCa3.1 channel blocker, was found to be safe and well tolerated for outpatients with sickle cell anemia in a phase III clinical trial (126). During the special COVID-19 era, it was administered to patients with severe respiratory insufficiency caused by COVID-19 in a phase II trial, which found senicapoc was associated with significantly lower oxygenation, a result that contradict the hypothesis (127). The imbalance of sodium transport by ENaCs is an important mechanism of ARDS pulmonary edema (106). Inhaled AP301 (TIP peptide), an activator of ENaCs, was first used to examine local and systemic safety in humans with ascending single doses in the year 2014 (128). This ‘first-in-human’ study found that doses up to 120 mg were well tolerated and safe for healthy male participants. The inhaled AP301 was primarily distributed to the lungs with very low exposure levels (128). Subsequently, the clinical effect of inhaled AP301 on extravascular lung water index was assessed in a phase IIa randomized controlled trial with mild to moderate ARDS, which indicated that inhaled AP301 only benefited the patients with sequential organ failure assessment scores ≥11 (129). A protocol for a Phase IIB clinical trial has been published to investigate the safety of AP301 and potential future efficacy endpoints in patients with ARDS (130).

Insufficient clinical studies indicate that there is still a significant gap between understanding the role of MSICs in the pathogenesis of VILI and identifying clinical therapeutic targets. This gap should prompt further research to accelerate the discovery of their potential clinical applications. The current *in vitro* studies primarily utilize a single cell population and apply linear stretching as an intervention method, which cannot simulate the human three-dimensional lung, composed of multiple cell types, nor the real movement of lung tissue during the breathing process. With the development of 3D organ printing technology, it is both necessary and possible to employ organoids to explore the comprehensive effects of mechanical stretching on MSICs in multicellular and three-dimensional lung tissue (131). The mechanisms of action of these inhibitors or agonists on MSICs are not fully understood, especially for the new member of MSICs (Piezo) and more in-depth research on the structural pharmacology of these drugs is suggested to conduct by the advanced techniques such as Cryo-electron microscopy (132). For drugs with promising prospects and strong safety profiles such as KCa3.1 channel inhibitors (133), TRPV4 antagonists (123) and ENaCs activator, it is possible to consider conducting preclinical studies and even clinical trials in special patient populations with caution, under the premise of ethical compliance.

This study met with some limitations. Firstly, *in vivo* studies on the role of MSICs in the occurrence of VILI, as well as the impact of drugs (formulations) on this role, are predominantly based on animal experiments. *In vitro* experiments that explore these issues are also mainly conducted using cells from animals, particularly rodents, although specific human cell populations such as AECs and PMVECs are used in some studies. Therefore, the current evidence is primarily derived from animals and their cell populations. Due to the species gap between humans and rodents, these findings cannot be directly extrapolated to humans in this review. Secondly, the mechanism, such as signal transduction pathways and pyroptosis, may only be interpreted in a single study, thus making the research evidence weak. Thirdly, due to inconsistencies in observed indicators and effect sizes, coupled with an insufficient number of existing literatures, there was no way in this study to pool the data for more robust evidence, which might induce conclusion with bias in a certain extent. Finally, clinical recommendations could not be formed in this study because of a paucity of clinical trials or preclinical trials.

## Conclusion

In conclusion, the emerging roles of MSICs in VILI have been concerned although they still have not been fully elucidated. The results indicated non physiological mechanical stretch stimulates MSICs to increase transcellular ion exchange and subsequently generates VILI through signal transmitting signaling pathways, which mediated inflammation, disruption of cell junctions, edema formation, oxygen reaction, pyroptosis, and the other pathogenesis. These findings make it possible to identify some potential therapeutic targets for the prevention of lung injury. However, the exact underlying mechanism needs further exploration, and more studies should be provoked.

## Data Availability

The original data presented in the study are included in the article. Further inquiries can be directed to the corresponding authors.
